# Impacts of a COVID-19 Educational Video: Evaluation of the Influence of Race, Gender, Political Affiliation, Study Major, and Age on Vaccine Acceptance among University Students

**DOI:** 10.3390/ejihpe13090126

**Published:** 2023-09-08

**Authors:** Audrey J. Lee, Tiffany T. Vu, Reina Marie Sanz, Myo-Kyoung Kim

**Affiliations:** Thomas J. Long School of Pharmacy, Pharmacy Practice, University of the Pacific, Stockton Campus, Stockton, CA 95211, USA; alee@pacific.edu (A.J.L.); t_vu17@u.pacific.edu (T.T.V.); r_sanz@u.pacific.edu (R.M.S.)

**Keywords:** SARS-CoV-2, COVID-19 vaccines, vaccine acceptance, vaccine hesitancy

## Abstract

Background: The World Health Organization (WHO) warns that vaccine hesitancy is an ongoing major global health threat. While vaccination against severe acute respiratory syndrome coronavirus (SARS-CoV-2) proves to be an effective strategy in protecting against the disease, vaccine hesitancy represents a major barrier to stopping the spread of the virus. Willingness for vaccination can be influenced by several factors, including education level and health literacy. Although several studies demonstrate the value of video educational programs in improving coronavirus disease 2019 (COVID-19) vaccine knowledge and acceptance, no studies to date have evaluated if race, gender, and other demographic factors impact the influence of an educational video on COVID-19 vaccine knowledge and hesitancy among university students in the United States (U.S.). Aims: This study was conducted to determine the impact of an educational video on U.S. university undergraduate students’ COVID-19 vaccine perception and acceptance. It also aims to evaluate whether demographic factors affect the influence of the video. Methods: An online survey was used to measure perceived understanding and acceptance of COVID-19 vaccines before and after viewing a video regarding the effectiveness and safety of COVID-19 vaccinations. The impact of demographic factors on the Video Influence Score was analyzed. Key results: After viewing the video, respondents’ (n = 285) perceived awareness and acceptance of COVID-19 vaccines significantly increased (*p* < 0.05). In addition, gender, political party affiliation, age, study major, and influenza vaccination history did not significantly impact the Video Influence Score (*p* > 0.05). However, African American/Black respondents (3.81 ± 4.24) were significantly more influenced by the video compared to respondents of other races (*p* < 0.05), such as White/Caucasian (1.91 ± 3.75), Hispanic/Latino (0.17 ± 3.67), Asian (0.29 ± 1.53), and Indigenous American (0.64 ± 2.52). Conclusions: This study suggests the potential impact of an educational video on COVID-19 vaccine perception and acceptance among university students. Despite limitations such as a modest survey response rate, this study provides valuable insight concerning the influential factors affecting vaccine acceptance in diverse student populations. Future studies are warranted to explore how student response to vaccine educational videos may vary depending on students’ racial and cultural backgrounds. Implications: A targeted educational video to promote vaccine acceptance is a valuable tool for public health campaigns to combat vaccine hesitancy. The study also highlights the importance of tailoring interventions to specific demographic groups such as considering racial factors to maximize the impact of educational interventions on vaccine attitudes.

## 1. Introduction

In late 2019, a new strain of coronavirus, SARS-CoV-2, was identified in Wuhan, China [1]. The virus caused COVID-19, which spread internationally, leading to the WHO declaration of the COVID-19 global pandemic on 11 March 2020 [2]. At the start of the pandemic, the U.S. and countries within the European Union and Asia implemented widespread mitigation policies to curb the global spread of the virus [3,4,5,6,7]. Among these policies were stay-at-home orders, mask mandates, and social distancing [3,4,5,6,7,8,9]. Beginning in December 2021, the U.S. Food and Drug Administration (FDA) issued emergency use authorization (EUA) for the Pfizer-BioNtech COVID-19 vaccine for patients ages 16 and over and for the use of the Moderna COVID-19 vaccine for adult patients [10,11,12]. By late February 2021, the FDA issued a third EUA for use of the Janssen COVID-19 vaccine in adult patients [10,13]. COVID-19 vaccines and subsequent boosters have been proven to be effective in preventing viral transmission among the public, mitigating severe symptoms, and minimizing hospitalizations [14,15].

The WHO [16] considers vaccine hesitancy or a “delay in acceptance or refusal of vaccination despite availability of vaccination services” as a major threat to global health [17]. Addressing vaccine hesitancy is crucial for mitigating a rise in COVID-19 cases, severe illnesses, and hospitalizations. This is critical in the context of rapid transmissions of SARS-CoV-2, the emergence of SARS-CoV-2 Omicron subvariants [18], and the lifting of masking and social distancing restrictions.

Although U.S. educational institutions have resumed in-person learning since 2021, several reports suggested that vaccine hesitancy towards COVID-19 boosters continues to remain high among American university students and young adults in 2023 [19,20,21,22,23,24,25,26]. In June 2023, the CDC COVID Data Tracker [27] demonstrated that only 20.5% of the U.S. population, age 18 years or older, had received an updated COVID-19 vaccine (bivalent) booster. Studies surveying the extent of vaccine hesitancy among university students in the U.S. and worldwide have found that approximately 14–45% of university students have demonstrated COVID-19 vaccine hesitancy [19,20,21]. While a lower level of education is associated with increased vaccine hesitancy [22,28], undergraduate college students still represent an important population for targeting COVID-19 vaccine promotion efforts [19,20,21,29]. Compared to graduate students, the undergraduate student population had a higher reported vaccine hesitancy [19]. Implementation of educational initiatives is important to reduce vaccine hesitancy and improve vaccination rates, which could decrease COVID-19 disease transmission, severe complications, hospitalizations, and deaths [28,29].

The use of a video-based educational tool has been shown to provide standardized content and enhance comprehension among participants with low literacy levels [30]. Reasons associated with COVID-19 vaccine hesitancy include lack of trust in COVID-19 vaccines, fear of vaccine side effects, and lack of knowledge about the COVID-19 vaccine [22,23,24,25,28,29,30]. Studies have demonstrated the positive effect of videos in increasing patient and caregiver knowledge and therapeutic decision-making [30,31,32,33,34]. Educational videos may also increase patients’ understanding of the risks and benefits of treatment options, address psychological barriers associated with COVID-19 vaccine hesitancy [30], and empower patients to adhere to governmental directives [31,32,33,35]. By improving knowledge and clarifying misconceptions related to COVID-19 vaccines, a targeted video educational program may increase COVID-19 vaccine acceptance and, in turn, vaccination rates.

However, few studies have evaluated the impact of an educational video on COVID-19 vaccine hesitancy or acceptance. In addition, few have investigated how determinants, such as race and gender, play a role [22,23,24,28,30,35]. Jensen et al. demonstrated that campaign messages via online videos can serve as effective tools for improving perceptions about vaccines and addressing knowledge gaps about the COVID-19 vaccine [30]. However, the study did not evaluate the influence and impact of educational videos on university students, nor the impact of gender, race, and other demographic factors on vaccine hesitancy. Although several studies [22,23,24,28,30,35] demonstrate the value of video educational programs in improving COVID-19 vaccine knowledge and acceptance, no studies to date have evaluated if race, gender, and other demographic factors impact the influence of an educational video on COVID-19 vaccine knowledge and hesitancy among university students in the U.S.

Therefore, the primary purpose of this study is to evaluate the impact of a COVID-19 educational video on university undergraduate students’ perception and acceptance of the COVID-19 vaccines. A secondary objective is to assess the influence of race, gender, age, political party affiliation, study majors, and previous influenza immunization history on students’ perception and acceptance of COVID-19 vaccines and their Video Influence Scores.

## 2. Materials and Methods

This cross-sectional study used an anonymous online survey, which was completed by a random sampling of U.S. university or college students. Between June and August 2021, responses were collected through the online questionnaire platform, Survey Monkey.

### 2.1. Participants

Survey participants were included if they were at least 18 years of age, a U.S. university or college student, had no history of receiving a COVID-19 vaccine, and had no contraindications for receiving a COVID-19 vaccine. Respondent participation was incentivized with gift cards upon completion of the survey. Over 200 colleges and universities were randomly selected from the U.S. News 2021 “Best National Universities” ranking list. From these selected schools, randomly selected student organizations were sent an email using contact information publicly available on school websites. The emails included a description of the study, information regarding incentives, the inclusion criteria, an online consent form, an educational video, and a survey link. The email contacts were also asked to distribute the information to members of the student organizations. Participant recruitment was also conducted through physical flyer distribution and social media, including Facebook. Prior to starting the questionnaire, potential survey respondents completed online eligibility screening questions and were provided with the study description and online consent form. Survey respondents who agreed to participate in the study were then instructed to complete the online survey.

### 2.2. Survey Instrument

A 7.4 min educational video in English was created by study investigators to provide background information on the epidemiology and complications of COVID-19, as well as the risks and benefits of receiving the COVID-19 vaccine. An online anonymous perception survey was then administered in English via Survey Monkey. The survey consisted of six demographic questions, nineteen Likert scale questions, and three validity questions. Demographic variables included participant age, gender, college major, race, political party affiliation, and the frequency of obtaining the influenza vaccine in the last five years. To safeguard respondents’ free will, participants were given the option to choose “prefer not to answer” for any questions. The selection of “prefer not to answer” was reported as “no response”.

COVID-19 vaccine perception and acceptance before and after watching the video were measured using six survey items using Likert scale questions (1 = “strongly disagree” to 5 = “strongly agree”): (1) knowledge of the side effects of COVID-19, (2) willingness to receive a COVID-19 vaccine, (3) belief in the benefits of vaccinations, (4) awareness of risks for contracting COVID-19, (5) fear of potential side effects from COVID-19 vaccines, and (6) belief that COVID-19 vaccines will prevent infection. Likert scale questions were also used to assess the motivating factors for obtaining the COVID-19 vaccine and the quality of the educational video.

Three additional questions were added as validity measures to identify potential reckless survey responses from participants who did not watch the educational video or were primarily motivated by the financial incentives of the survey. These validity questions provided evidence that the participants had watched the entire video by testing participant knowledge of the video length and content, such as the age range of individuals providing testimonies in the video. Survey respondents with incorrect responses to any of these validity questions were excluded from analysis.

### 2.3. Measurements

Vaccine acceptance was assessed by determining the number of participants who indicated that they “strongly agreed” or “agreed” with the survey item that they were “willing to receive a COVID-19 vaccine”.

The Likert scale questions addressing student beliefs and attitudes towards the COVID-19 vaccine were aggregated and further evaluated based on the Total Perception Scores determined before and after video viewing. The Total Perception Scores were calculated from the sum of the scores obtained from six survey items that addressed vaccine perception and acceptance of the COVID-19 vaccine. For the perception assessment question addressing participant fear of COVID-19 vaccine side effects, a reversed rubric scoring system (1 = “strongly agree” to 5 = “strongly disagree”) was used in the Total Perception Score calculation since this item measured negative perception about the COVID vaccine. Video Influence Scores were determined by calculating the difference between the Total Perception Scores before and after video viewing.

### 2.4. Statistical Analysis

An a priori power analysis was conducted using G*Power version 3.1.9.7 [36] to determine the minimum sample size required to test the difference between scores before and after viewing the educational video. Using a t-test, a sample size of 150 was calculated to achieve 80% power for detecting a medium effect (effect size = 0.2) with a significance level of α = 0.05.

The Statistical Package for the Social Sciences (SPSS), version 27.0, was used for statistical analysis. Descriptive statistics were used to summarize the demographic data, vaccination motivators, and quality of education. Norman [37] and Sullivan [38] reported the suitability of employing parametric data analysis for data collected using Likert scales. Subsequently, the paired t-tests measured the difference in participants’ responses before and after watching the educational video. Independent t-tests were used to assess the effects of gender and political party affiliation on the Total Perception Scores and Video Influence Scores. ANOVA and post hoc pairwise comparisons using Tukey’s honestly significant difference (HSD) test were conducted to assess the potential impact of race and area of study on participants’ Total Perception Scores and Video Influence Scores. Pearson correlation tests were also used to measure the relationship between the following pairs: age and Total Perception Scores, age and Video Influence Scores, number of influenza vaccinations in the past five years and Total Perception Score, and number of influenza vaccinations in the past five years and Video Influence Score.

## 3. Results

Recruitment emails were sent to 5986 student organizations, and 515 students submitted a survey response. Of the 515 survey respondents, 497 met the inclusion criteria, while 18 were excluded because they declined to participate in the study or did not meet the inclusion criteria. Due to incorrect responses to the validity questions, 212 respondents were further excluded, resulting in 285 respondents with valid survey responses that were included in the data analysis.

### 3.1. Demographics

The demographics are summarized in Table 1. Most survey respondents included in the analysis (n = 285) were male (59%) with a mean age of 23.9 ± 4.27. Respondents were primarily White/Caucasian (34.7%) or Hispanic (32.6%). A total of 41.4% of respondents were affiliated with the Republican Party while 38.6% were affiliated with the Democratic Party. Most students identified their major of study as engineering and science (45.3%) or social science (37.5%). A total of 84.2% of participants reported receiving the influenza vaccine at least once in the previous five years.

### 3.2. Effects of Video

Table 2 describes undergraduate students’ COVID-19 vaccine perception and acceptance before and after viewing the educational video.

**Figure 1:**
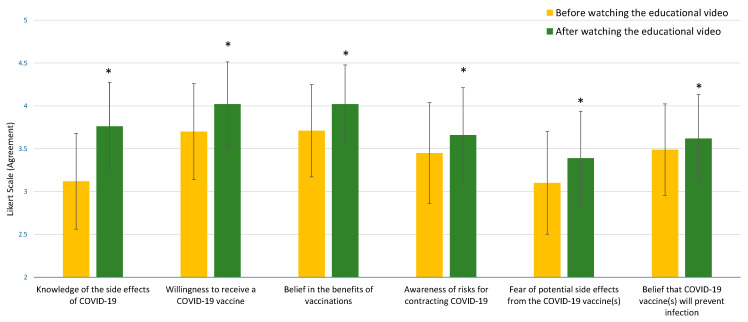
COVID-19 vaccine perception and acceptance before and after watching the video (n = 285). Asterisks (*) indicate significant differences (*p* < 0.001) before and after watching the video.

All scores of the six survey items that measured COVID-19 vaccine perception and acceptance were significantly improved after respondents watched the educational video as shown in Figure 1 (*p* < 0.001), with the greatest effect of the video associated with an increased understanding of COVID-19 vaccine side effects. Compared to baseline, a significantly higher percentage of respondents indicated that they were at risk for contracting COVID-19 (56.8% vs. 62.4%) and that the COVID-19 vaccines were protective (55.1% vs. 59.3%) after watching the video. Overall, significantly more respondents were willing to receive a COVID-19 vaccine after viewing the video (67.0% vs. 75.7%). Overall vaccine hesitancy, based on the percentage of respondents that “strongly disagreed” or “disagreed” that they were “willing to receive a COVID-19 vaccine,” decreased from 13.4% to 7.5% after viewing the video. For questions focused on the quality of the educational video, approximately 72% of respondents (205/285) agreed that the educational video helped them understand COVID-19 vaccine benefits, and 75.8% (216/285) agreed that they would recommend the video to others.

### 3.3. Impact of the Video and Demographic Factors: Race, Study Majors, Gender, Political Affiliation, Age, and Influenza Vaccine History

The influence of demographic factors (i.e., race, study majors, gender, and political affiliation) on Total Perception Scores before viewing the video, Total Perception Scores after viewing the video, and Video Influence Scores were summarized in Table 3.

**Figure 2:**
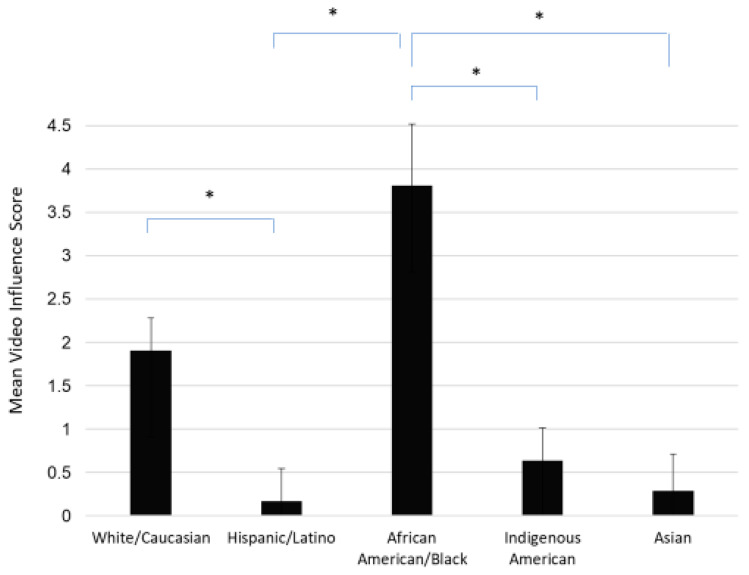
Video Influence Scores (difference between Total Perception Scores before and after viewing video). Asterisks(*) indicate significant differences (*p* < 0.05) between each pair.

As shown in Figure 2, the Video Influence Scores were significantly different among different races (*p* < 0.01). Prior to viewing the video, Asian respondents had the highest Total Perception Score (23.06 ± 3.77) compared to other races, whereas African American/Black respondents had the lowest score (18.61 ± 3.89). Interestingly, after respondents watched the video, the Total Perception Score was not significantly different in each of the racial groups, as shown in Table 3 (*p* > 0.05). However, the video’s influence on vaccine perception was significant (*p* < 0.01). In other words, African American/Black respondents (3.81 ± 4.24) were most significantly influenced by the video compared to White/Caucasian (1.91 ± 3.75), Hispanic/Latino (0.17 ± 3.67), Asian (0.29 ± 1.53), and Indigenous American respondents (0.64 ± 2.52).

As depicted in Table 2, the Total Perception Scores before and after video exposure, along with the Video Influence Scores, demonstrated no statistically significant difference (*p* > 0.05) across the diverse academic disciplines. In terms of gender comparison, females displayed a significantly elevated Total Perception Score (22.25 ± 3.38) after watching the educational video, in contrast to males (21.27 ± 3.08) (*p* = 0.013). However, the Video Influence Scores of both females and males were not significantly different (*p* = 0.75).

Democrats had a significantly higher Total Perception Score before watching the video (21.51 ± 3.56) compared to Republicans (20.32 ± 2.89, *p* = 0.006). However, while Democrats continued to have a higher perception score of the COVID-19 vaccine (22.3 ± 3.47) after watching the video compared to Republicans, (20.91 ± 3.22, *p* = 0.002), there was no statistically significant difference (*p* = 0.62) in the Video Influence Scores between both political parties.

A negative correlation (r = −0.14) between age and Total Perception Score was determined before watching the video (*p* = 0.02) while no correlation was shown after watching the video (*p* = 0.10). However, the Video Influence Score was not statistically correlated with age (*p* = 0.93). In addition, the frequency of respondents who received an influenza vaccine in the last five years was strongly correlated with the Total Perception Scores before and after watching the educational video (*p* < 0.01) but was not strongly correlated with the Video Influence Score (*p* = 0.41).

### 3.4. Motivating Factors Affecting COVID-19 Vaccine Willingness

Table 4 summarizes the motivating factors for receiving the COVID-19 vaccine. Over 80% of the respondents indicated that they were motivated to receive the COVID-19 vaccine because they wanted to protect others from acquiring a COVID-19 infection and to stop the spread of the COVID-19 infection. In comparison, approximately 72% specified that they wanted to protect themselves against COVID-19 infection and related complications. Other less common motivating factors affecting COVID-19 vaccine willingness included shortening the duration of the COVID-19 pandemic (67% of respondents), school mandates to receive the COVID-19 vaccine(s) (49% of respondents), and desire to gather indoors without masks (57% of respondents).

## 4. Discussion

According to the WHO COVID-19 database [39], the U.S. continues to have one of the highest numbers of COVID-19 cases globally as of March 2023 [40]. In the U.S., the CDC [18] reports that the widely transmissible omicron variant is the most prevalent variant as of February 2023. According to a study by Plumb et al. [14] during the Omicron-predominate era, mRNA COVID-19 vaccination may prevent COVID-19-associated hospitalizations in patients with a SARS-CoV-2 infection by approximately 35% after a second mRNA vaccine dose, and 68% after the first vaccine booster. Further studies [15] report that a third mRNA vaccine dose 14 days after administration prevented COVID-19-related hospitalizations from the Omicron variant by 90% or by 78% four or more months after booster administration. These findings underscore the importance and need for receiving the primary mRNA two-shot COVID-19 vaccine series along with at least one booster dose to obtain protection against COVID-19 infection.

This study demonstrated that an educational video could influence COVID-19 vaccine perception in unvaccinated university students. A significantly higher percentage of students reported that they “believe[d] in the benefit of vaccinations” and “believe[d] that COVID-19 vaccines will protect them” after watching the video. A study conducted in Saudi Arabia found increased knowledge regarding COVID-19 vaccines after a video-based education intervention in adults [41]. The study surveyed 500 Saudi Arabian adults, 250 of whom watched two videos about COVID-19 vaccines. The group who received the video intervention showed a significantly higher proportion of good knowledge regarding general vaccine mechanisms (87.4% vs. 43.5%, *p* < 0.001), information about vaccines (73.1% vs. 35.7%, *p* < 0.001), vaccine formation process (7.9% vs. 5.9%, *p* < 0.001), availability of COVID-19 vaccine in Saudi Arabia (28.5% vs. 7.8%, *p* < 0.001), and overall knowledge (71.9% vs. 4.3%, *p* < 0.001). The influence of a COVID-19 educational video was also reported by Kaim et al. [35] in older non-University adult patients. This study surveyed 503 Israeli adults (mean age 43.81 ± 15.84) and demonstrated a significant increase in perceived COVID-19 vaccine knowledge (*p* < 0.001) after the participants watched the video. The study also found an increase in the understanding of the importance (*p* = 0.002) and protection (*p* < 0.001) of COVID-19 vaccines. The impact of the video was found to be less significant in respondents who were vaccine-hesitant.

In this present study, student willingness to receive a COVID-19 vaccine was approximately 67% prior to viewing the educational video. These findings are consistent with the Gurley et al. [25] study, which reported a 65% COVID-19 vaccine acceptance for an unvaccinated Massachusetts college cohort (n = 105). Our study further demonstrated that the educational video significantly increased student acceptance of the COVID-19 vaccine from 65% to 76%. Another study [30] that was not limited to university students similarly demonstrated the positive influence of educational videos on vaccine willingness.

This study further demonstrates the presence of racial disparities among student cohorts in COVID-19 vaccine perception at baseline. This study uniquely demonstrated that African American/Black students were most influenced by the educational video compared to Indigenous American, Hispanic/Latino, or Asian students. While other studies have not directly evaluated video impact on COVID-19 vaccine hesitancy, several studies have reported that COVID-19 vaccine hesitancy differs among racial groups [25,27,42,43]. In a study by Elliot and Yan [19] the odds ratio (OR) of COVID-19 vaccine hesitancy was higher in American Indian (OR 2.92) or African American/Black (OR 2.16) students and least likely to be reported in Asian students (OR 0.58, *p* < 0.001) when compared to White/Caucasian students. Others report similar findings of COVID-19 vaccine hesitancy in African Americans compared to other racial groups [22,25,42,43,44,45]. None of these studies investigated the causes or cultural differences that influenced vaccination hesitancy.

This study also illustrates the influence of political affiliation and previous history of receiving the influenza vaccine on COVID-19 vaccine acceptance. As shown in the studies by Dhanani et al. [44] and Lennon et al. [45], survey respondents who reported a Democratic Party affiliation demonstrated a higher COVID-19 vaccine perception score and were more willing to receive the COVID-19 vaccine prior to watching the video compared to Republican Party respondents. This study demonstrated that participants who had a previous history of receiving the influenza vaccine also had higher COVID-19 vaccine perception scores and were more willing to receive a COVID-19 vaccine prior to watching the video, particularly if they reported obtaining the influenza vaccine annually in the past five years. However, respondents’ political affiliation and history of receiving the influenza vaccine did not influence their willingness to receive a COVID-19 vaccine after respondents watched the video, since the respondents’ overall COVID-19 vaccine acceptance remained unchanged from baseline.

Findings from this study further demonstrated that female students have significantly higher Total Perception Scores both before and after watching the video. A Wotring et al. study [21] also reported that female participants had a greater willingness to receive a COVID-19 vaccine compared to male participants. Conversely, other studies [22,44] have described that females were significantly more hesitant to receive the COVID-19 vaccine compared to males. One plausible reason for the differing results is that sources of health information may vary across student groups [27]. The findings from the Elliot and Yan study [19] describe that vaccine-hesitant respondents were more likely than vaccine-accepting respondents to rely on their “employers” (6.4% vs. 4.45%) or healthcare providers (5% vs. 2.5%) compared to public health agencies (30.7% vs. 43%) as a COVID-19 information. 

This study had some methodological limitations. The survey response rate based on the percentage of university students willing to watch the video and participate in the study was approximately 8.6%, a relatively low response rate. It is possible that survey respondents were not a representative sample of the U.S. university student population, since the study excluded students who previously received COVID-19 vaccine(s). Additionally, only adult participants older than 18 years old were included in the study. Minors were excluded from the study because they required parental consent. Thus, the results of the study did not apply to university students younger than 18 years old. Future directions could involve an assessment of other demographic factors, such as geographical differences, comorbidities, and academic performance, in relation to COVID-19 vaccine acceptance. The causes and cultural differences that influence vaccine hesitancy and the impact of educational videos on COVID-19 vaccine adherence in a “real world” practice setting could be further investigated.

## 5. Conclusions

An educational video had a significant effect on the perception and acceptance of COVID-19 vaccines among university students. This study suggests valuable insight concerning the influential factors affecting vaccine acceptance in student populations. The findings of this study support the use of educational videos to promote vaccine acceptance among university students for future public health endeavors. It also necessitates the implementation of such interventions to reach specific demographic groups who had been systemically overlooked in the past, such as African Americans, who were found to be most positively impacted by the educational videos. The targeting of video educational interventions may help maximize their impact on student vaccine attitudes. Future studies should explore how racial, geographical, and cultural differences influence student responses to educational videos.

## Figures and Tables

**Table 1:** Survey respondent demographics (n = 285).

Demographics	Number of Participants (%)
**Gender**	
Female	117 (41.1%)
Male	167 (58.6%)
Non-binary gender	1 (0.4%)
**Age**	
18–21	76 (26.7%)
22–23	86 (30.2%)
24–25	63 (22.1%)
>25	60 (21.1%)
**Race**	
White/Caucasian	99 (34.7%)
Hispanic/Latino	93 (32.6%)
African American/Black	36 (12.6%)
Indigenous American	36 (12.6%)
Asian	17 (6.0%)
Pacific Islander	0 (0%)
Other/No response	4 (1.4%)
**Political Affiliation**	
Republican	118 (41.4%)
Democrat	110 (38.6%)
Other/No response	57 (2.0%)
**Major**	
Engineering and Sciences	129 (45.3%)
Social Sciences	107 (37.5%)
Visual and Performing Arts	25 (8.8%)
Health Sciences	14 (4.9%)
Other/No response	10 (3.5%)
**Number of influenza vaccines received in the last 5 years**	
0	35 (12.3%)
1	42 (14.7%)
2	91 (31.9%)
3	58 (20.4%)
4	18 (6.3%)
5	31 (10.9%)
Other/No response	10 (3.5%)

**Table 2:** COVID-19 vaccine perception and acceptance before watching the video (n = 285).

		Before Watching the Video	After Watching the Video	*p*-Value
**Knowledge of the side effects of COVID-19 infection**	Strongly agree or agree	118 (41.4%)	188 (66.0%)	
	Neither agree nor disagree	84 (29.5%)	66 (23.2%)	
	Strongly disagree or disagree	83 (29.1%)	31 (10.9%)	
	Mean ± SD of Likert Scores	3.12 ± 1.18	3.76 ± 1.03	<0.001
**Willingness to receive a COVID-19 vaccine**	Strongly agree or agree	191 (67.0%)	216 (75.7%)	
	Neither agree nor disagree	56 (19.6%)	48 (16.8%)	
	Strongly disagree or disagree	38 (13.4%)	21 (7.5%)	
	Mean ± SD of Likert Scores	3.70 ± 1.12	4.02 ± 0.99	<0.001
**Belief in the benefits of vaccinations**	Strongly agree or agree	177 (62.1%)	216 (75.8%)	
	Neither agree nor disagree	65 (22.8%)	48 (16.8%)	
	Strongly disagree or disagree	43 (15.1%)	21 (7.4%)	
	Mean ± SD of Likert Scores	3.71 ± 1.08	4.02 ± 0.92	<0.001
**Awareness of risks of contracting COVID-19**	Strongly agree or agree	162 (56.8%)	178 (62.4%)	
	Neither agree nor disagree	59 (20.7%)	61 (21.4%)	
	Strongly disagree or disagree	64 (22.4%)	46 (16.2%)	
	Mean ± SD of Likert Scores	3.45 ± 1.18	3.66 ± 1.10	<0.001
**Fear of potential side effects from the COVID-19 vaccine(s)**	Strongly agree or agree	121 (42.5%)	148 (51.9%)	
	Neither agree nor disagree	75 (26.3%)	78 (27.4%)	
	Strongly disagree or disagree	89 (31.2%)	59 (20.7%)	
	Mean ± SD of Likert Scores	3.10 ± 1.20	3.39 ± 1.09	<0.001
**Belief that COVID-19 vaccine(s) will prevent infection**	Strongly agree or agree	157 (55.1%)	169 (59.3%)	
	Neither agree nor disagree	76 (26.7%)	82 (28.8%)	
	Strongly disagree or disagree	52 (18.2%)	34 (11.9%)	
	Mean ± SD of Likert Scores	3.49 ± 1.07	3.62 ± 1.03	<0.001
**Total Perception Score**	Mean ± SD of Likert Scores	20.37 ± 3.75	21.69 ± 3.24	<0.001

**Table 3:** Total Perception Scores before and after viewing video and Video Influence Scores (difference in Total Perception Scores before and after viewing video).

	Total Perception Scores before Viewing Video	Total Perception Scores after Viewing Video	Video Influence Scores
**Total**	20.37 ± 3.75	21.69 ± 3.24	1.32 ± 3.72
**Race**			
White/Caucasian	19.91 ± 4.04 ^A^	21.82 ± 3.26	1.91 ± 3.75 ^D^
Hispanic/Latino	20.86 ± 3.05 ^B^	21.03 ± 2.87	0.17 ± 3.67 ^D,E^
African American/Black	18.61 ± 3.89 ^B,C^	22.42 ± 3.51	3.81 ± 4.24 ^E,F,G^
Indigenous American	20.5 ± 3.57	21.14 ± 3.14	0.64 ± 2.52 ^F^
Asian	23.06 ± 3.77 ^A,C^	23.35 ± 3.90	0.29 ± 1.53 ^G^
**Major**			
Health Sciences	20.86 ± 3.48	22.14 ± 2.21	1.29 ± 4.80
Engineering and Sciences	20.28 ± 3.98	21.92 ± 3.34	1.64 ± 4.00
Social Sciences	19.93 ± 3.62	21.14 ± 3.16	1.21 ± 3.65
Visual and Performing Arts	20.76 ± 2.39	21.12 ± 2.70	0.36 ± 2.22
**Gender**			
Male	20.01 ± 3.63	21.27 ± 3.08 ^H^	1.26 ± 3.48
Female	20.83 ± 3.85	22.25 ± 3.38 ^H^	1.42 ± 4.07
**Political affiliation**			
Republican	20.32 ± 2.89 ^I^	20.91 ± 3.22 ^J^	0.58 ± 2.72
Democrat	21.51 ± 3.56 ^I^	22.3 ± 3.47 ^J^	0.79 ± 3.53

Each mean ± SD with any superscript indicates that it has a significant difference (*p* < 0.05) between each pair, marked as the same superscript (e.g., A, B).

**Table 4:** Motivating factors identified by respondents (n = 285) for receiving the COVID-19 vaccines.

	Strongly Agree or Agree	Neither Agree Nor Disagree	Strongly Disagree or Disagree	Mean ± SD of Likert Scores
To protect the people around me and stop the spread of COVID-19 infection	230 (80.7%)	36 (12.6%)	19 (6.7%)	4.91 ± 0.93
To protect oneself from getting a COVID-19 infection and its complications	207 (72.3%)	53 (18.6%)	26 (9.1%)	3.90 ± 0.97
To shorten the duration of the COVID-19 pandemic	191 (67.0%)	66 (23.2%)	28 (9.9%)	3.91 ± 1.05
My school requires that I obtain the COVID-19 vaccine	162 (56.9%)	61 (21.4%)	62 (21.7%)	3.50 ± 0.93
To gather indoors with other people without masks	139 (48.8%)	64 (22.5%)	82 (28.7%)	3.16 ± 1.28

Participants were allowed to identify more than one motivating factor.

## Data Availability

The data presented in this study are available upon request.
